# Lipid laden macrophages and electronic cigarettes in healthy adults

**DOI:** 10.1016/j.ebiom.2020.102982

**Published:** 2020-09-10

**Authors:** Peter G. Shields, Min-Ae Song, Jo L. Freudenheim, Theodore M. Brasky, Joseph P. McElroy, Sarah A. Reisinger, Daniel Y. Weng, Rongqin Ren, Thomas Eissenberg, Mark D. Wewers, Konstantin Shilo

**Affiliations:** a Comprehensive Cancer Center, The Ohio State University and James Cancer Hospital, 460W. 10th Avenue, 9th Floor, Suite D920, Columbus, OH 43210-1240, United States; b Division of Environmental Health Science, College of Public Health, The Ohio State University, Columbus, United States; c Department of Epidemiology and Environmental Health, School of Public Health and Health Professions, University at Buffalo, Buffalo, NY, United States; d Center for Biostatistics, Department of Biomedical Informatics, The Ohio State University, Columbus, OH, United States; e Department of Pathology, Ohio State University Wexner Medical Center, Columbus, OH, United States; f Center for the Study of Tobacco Products, Department of Psychology, Virginia Commonwealth University, Richmond, VA, United States; g Pulmonary and Critical Care Medicine, Department of Internal Medicine, The Ohio State University, Columbus, OH, United States

**Keywords:** Bronchoscopy, Nicotine, Vaping, Cytokines, EVALI

## Abstract

**Background:**

An outbreak of E-cigarette or Vaping Product Use-Associated Lung Injury (EVALI) with significant morbidity and mortality was reported in 2019. While most patients with EVALI report vaping tetrahydrocannabinol (THC) oils contaminated with vitamin E acetate, a subset report only vaping with nicotine-containing electronic cigarettes (e-cigs). Whether or not e-cigs cause EVALI, the outbreak highlights the need for identifying long term health effects of e-cigs. EVALI pathology includes alveolar damage, pneumonitis and/or organizing pneumonia, often with lipid-laden macrophages (LLM). We assessed LLM in the lungs of healthy smokers, e-cig users, and never-smokers as a potential marker of e-cig toxicity and EVALI.

**Methods:**

A cross-sectional study using bronchoscopy was conducted in healthy smokers, e-cig users, and never-smokers (*n* = 64). LLM, inflammatory cell counts, and cytokines were determined in bronchial alveolar fluids (BAL). E-cig users included both never-smokers and former light smokers.

**Findings:**

High LLM was found in the lungs of almost all smokers and half of the e-cig users, but not those of never-smokers. LLM were not related to THC exposure or smoking history. LLM were significantly associated with inflammatory cytokines IL-4 and IL-10 in e-cig users, but not smoking-related cytokines.

**Interpretation:**

This is the first report of lung LLM comparing apparently healthy smokers, e-cig users, and never-smokers. LLM are not a specific marker for EVALI given the frequent positivity in smokers; whether LLMs are a marker of lung inflammation in some e-cig users requires further study.

**Funding:**

The National Cancer Institute, the National Heart, Lung, and Blood Institute, the Food and Drug Administration Center for Tobacco Products, the National Center For Advancing Translational Sciences, and Pelotonia Intramural Research Funds

**Research in Context**

**Evidence before this study**

Electronic cigarettes (e-cigs) deliver nicotine and flavours via heated e-liquid carriers, including propylene glycol and vegetable glycerin. The recent outbreak of E-cigarette or Vaping Product Use-Associated Lung Injury (EVALI) is associated with e-liquids containing tetrahydrocannabinol and vitamin E acetate. However, a subset of cases reported exclusive use of nicotine-containing e-cigs. EVALI has been characterized by features including diffuse alveolar damage, acute eosinophilic pneumonia, hypersensitivity pneumonitis, and lipoid pneumonia. As the cytopathology of acute EVALI showed the presence of pulmonary foam macrophages in some cases, some have suggested lipid-laden macrophages (LLM) are markers of vaping associated lung injury.

**Added value of this study**

Understanding the toxicity of e-cigs on the lung is urgently needed. The examination of LLM as a diagnostic or susceptibility marker for EVALI has potential. In this cross-sectional bronchoscopy study of healthy smokers, e-cig users, and never-smokers, LLM were found in most smokers, in about half of e-cig users, and almost no never-smokers. LLM were uniquely related to IL-4 and IL-10 in e-cig users, but not in smokers and never-smokers.

**Implications of all the available evidence**

This is the first report of LLM in healthy participants, examining smokers, e-cig users, and never-smokers. We found that LLM are not specific to EVALI; LLM are present in healthy smokers and some e-cig users. For those e-cig users, LLM were associated with some inflammatory cytokines, and so may be a marker of health effects in a subset of e-cig users.

Alt-text: Unlabelled box

## 1 Introduction

The recent 2019 outbreak of E-cigarette or Vaping Product Use-Associated Lung Injury (EVALI) has mostly implicated vaping with oils containing tetrahydrocannabinol (THC) and other constituents such as vitamin E acetate [1]. However, there is a subset of cases who reported exclusive use of nicotine-containing electronic cigarettes (e-cigs) [2]. EVALI pathology of lung injuries include acute fibrinous pneumonitis, diffuse lung injury, organizing pneumonia, and the presence of foamy macrophages (lipid-laden macrophages [LLM]), and in some cases also feature as a variant of lipoid pneumonia with LLM [3], [4], [5], [6], [7].

E-cig use is increasing rapidly among youths, and e-cigs are commonly used by smokers intending to quit. E-cigs have been proposed as an alternative method for nicotine consumption that is considered by some to be a lower risk product but may have other health effects, as suggested by the outbreak of EVALI. E-cigs contain propylene glycol (PG) and vegetable glycerin (VG) and are different from vaping oils with THC. Both PG and VG are generally recognized as safe by the Food and Drug Administration as additives in foods and cosmetics. However, the pulmonary effects of aerosolized PG/VG and the value of LLM as an EVALI diagnostic or susceptibility marker are not well understood. E-cig toxicity investigations typically focus on an overlap with cigarette smoking disease pathways. Still, the recent EVALI outbreak indicates the need for a broader exploration of e-cig health effects. Whether there is value in LLM as an EVALI diagnostic or susceptibility marker is not clear, and it should be evaluated in healthy e-cig users, and be compared to cigarette smokers and never-smokers.

We conducted a cross-sectional study of healthy adult smokers, e-cig users, and never-smokers who underwent bronchoscopy to assess toxicity. We examined differences both related and unrelated to cigarette smoking. We report here on differences in the presence of lung LLM and inflammatory cytokines in these healthy young adults.

## 2 Methods

### 2.1 Participants and study design

Sixty-four healthy participants were recruited between 2015 and 2019 through the Ohio State University (OSU) Study Search website, a participant registry from the OSU Tobacco Centers of Regulatory Science, local print media, television, radio, and Craigslist. Eligible participants were healthy adults aged 21–45, willing to undergo bronchoscopy. This study was approved by the OSU Institutional Review Board (OSU-2015C0088) and registered on Clinicaltrials.gov (NCT02596685). All participants provided informed consent prior to participation.

Participants were never-smokers (*n* = 28) who had smoked <100 cigarettes in their lifetime, and had not used an e-cig or cigarette for >1 year prior to the study; e-cig users (*n* = 13) who had puffed nicotine-containing e-cigs daily for >1 year and had no cigarette smoking for >5 months; and, exclusive active cigarette smokers (*n* = 23) who had current use of ≥10 cigarettes/day [cigs/day] for >6 months, and had not used an e-cig for at least one year. Reported smoking status was confirmed by saliva or urine cotinine using a NicAlert kit (Nymox Pharmaceutical Corporation, St. Laurent, QC, Canada) (tobacco users and e-cig users were defined at test level >1). Participants were excluded if they had increased risk of complications from the bronchoscopy procedure or had an immune system disorder requiring medication, reported a diagnosis of pulmonary disease (e.g., asthma within the prior 5 years, acute bronchitis within 1 year, COPD, chronic bronchitis, and restrictive lung diseases), reported a diagnosed history of kidney or liver diseases, or other medical disorders that would affect biomarker data (see full list of exclusions in Supplementary). Participants were also excluded if they had undergone general anesthesia during the prior 12 months, used any inhalant medications, had allergies to study medications (Lidocaine, Cetacaine Versed, or Fentanyl), had undergone a bronchoscopy or any other lung procedure within the prior 6 months, had regularly smoked marijuana in the past (>10 times), smoked marijuana within 3 months prior to the study procedure (self-reported), had other combustible tobacco use within 3 months prior to the procedure, or were pregnant (determined by urine pregnancy test prior to bronchoscopy).

### 2.2 Bronchoscopy procedure

Participants first attended an orientation session where a trained clinical research assistant obtained informed consent and conducted a brief medical history. After confirming the inclusion and exclusion criteria, with physician review, a bronchoscopy visit was scheduled within 2 weeks. The bronchoscopy was performed under light intravenous sedation. For bronchoalveolar lavage (BAL), the bronchoscope was wedged into a subsegmental bronchus and using saline (20 ml), injected approximately 5–7 times recovering samples at each lavage. Subjects were sampled in the right or left lung based on a randomization protocol. Participants received $32 for the orientation session and $200 for the procedure.

### 2.3 Oil red o stain

Cytospin slides were made from 500 µl aliquots of BAL, centrifuged to transfer cells onto glass slides, and fixed. Fixed slides were rinsed with distilled water to remove fixative and stained in Oil Red O (Pfaltz and Bauer, Inc., Waterbury, CT) for 15 min, followed by a second distilled water rinsing. Slides were then stained in Hematoxylin (Thermo Scientific Richard-Allan, Waltham, MA) for 1 min, and blue sections were processed in ammonia water; after each step, slides were rinsed with tap water [8,9]. After the last tap water rinse, coverslips were placed on slides and then imaged. **Supplementary Figure 1** presents the validation of Oil Red O stain with adipose tissue and several BALs from smokers and never-smokers.

### 2.4 Lipid-laden macrophages (LLM)

The glass slides were used for whole slide imaging, and macrophage counts were performed on a representative area of the slide displaying at least 100 non-overlapping macrophages. A lipid-laden macrophage index (LLMI) was calculated as previously described [9]. The LLMI comprised the sum of the percentages of macrophages with >50% of the cytoplasm occupied by lipid multiplied by 2 and macrophages with <50% of the cytoplasm occupied by lipid, with a potential range from 0 to 200. Only cases with distinct red-brown cytoplasmic lipid droplets (LLMI >10) were considered positive.

### 2.5 Urinary biomarkers of exposure

*Trans-3-hydroxycotinine (3HC)* and *cotinine (COT)* were analyzed using trans-3-hydroxycotinine-d9 and cotinine-d9_,_ respectively, as internal standards by liquid chromatography tandem mass spectrometry (LC-MS/MS), as previously described [10]. Instrumentation LC-MS/MS analyses were performed with a Thermo Surveyor (Thermo Fisher Scientific, Waltham, MA), or Agilent 1200 HPLC interfaced (Agilent, Santa Clara, CA) to a Thermo-Finnigan TSQ Quantum Ultra triple-stage quadrupole mass spectrometer (Thermo Fisher Scientific, Waltham, MA) or using a Hewlett Packard 1090 HPLC (Hewlett Packard, Palo Alto, CA) interfaced with a Finnigan TSQ 7000 triple-stage quadrupole mass spectrometer (API-2 ion source) (ThermoQuest, San Jose, CA). Urine concentrations were normalized with creatinine. Molar sums were calculated for the nicotine equivalent (3HC plus COT) [10].

*Anatabine* was analyzed using anatabine-d4, as described previously [11]. LC/MS/MS analyses were carried out by positive ESI on a Thermo Scientific TSQ Vantage triple quadrupole mass spectrometer (Thermo Fisher Scientific) interfaced with a Dionex Ultimate 3000 Capillary HPLC.

*Nicotelline,* a tripyridine alkaloid present in tobacco and tobacco smoke, was used to detect recent cigarette smoking, which is not detected in e-cig users. It was measured using Nicotelline-d8 synthesized by Suzuki coupling of 2,4-diiodopyridine with 3-pyridine-d4-boronic as an internal standard as described previously [12]. LC-MS/MS analyses were carried out with a Thermo Accela UPLC pump, and Pal Autosampler interfaced to a Thermo Vantage triple-stage quadrupole mass spectrometer (Thermo Fisher Scientific) or with an Agilent 1200 HPLC (Agilent) interfaced to a Thermo-Finnigan TSQ Quantum Ultra triple-stage quadrupole mass spectrometer (Thermo Fisher Scientific).

*Urinary Propylene Glycol (1,2-propanediol)* was measured using 6 (±)−1,2-propanediol-d8 [CDN, d-1656]) as an internal standard, samples were analyzed by LC-MS/MS (Agilent 1290 Infinity II UPLC system). Eluted compounds were further separated and quantified through the coupled Agilent 6495 Triple Quadrupole equipped with an electrospray ion source.

*Carboxy-Tetrahydrocannabinol (THC)* was measured using gas Chromatography-Mass Spectrometry (GC–MS) by Mayo Clinic Laboratories (https://www.mayocliniclabs.com/test-catalog/Performance/8898).

### 2.6 BAL inflammatory cytokines

Levels of BAL inflammatory cytokines were measured using the V-PLEX Plus Proinflam Combo 10 panel (IFN-γ, IL-1β, IL-2, IL-4, IL-6, IL-8, IL-10, IL-12p70, IL-13, and TNF-α) from Meso Scale Discovery (Rockville, MD) according to the manufacturer's instructions. All samples were analyzed in duplicate, and averaged values were used for the statistical analysis. A mean intra-assy CVs for 10 cytokines was below 7%, and an inter-assay CV was below 15%, which fell within expected CVs by the manufacturer.

### 2.7 Statistical analysis

Data were analyzed in 7 batches, and batch effects were removed using ANOVA before the statistical analyses. Batch effects were removed by ANOVA with feature as the dependent variable and batch as the independent variable. The residuals from these models were used as the input features for analysis (Mann–Whitney between group pairs or Kruskal–Wallis across groups) for associations between batch-removed LLMI (continuous) and groups. Fisher's exact test was used to determine associations between LLM (positive and negative) and groups. Mann–Whitney were used for the association between LLM and cytokines. Spearman correlation was used for an association of batch-removed LLMI with smoking history, urinary biomarkers, inflammatory cell counts, and THC levels. Missing data were excluded before the statistical analysis. All statistical tests were two-sided and statistical significance was defined as comparison-wise *P*<0.05. To put into the context of multiple testing, across all comparisons herein (with the exception of demographics), a raw p-value of 0.05 corresponds to a Benjamini Hochberg false discovery rate of 0.16. Characteristics of study population (age, gender, and race) were not adjusted, as is standard for presenting sample characteristics.

## 3 Results

**Fig. 1:**
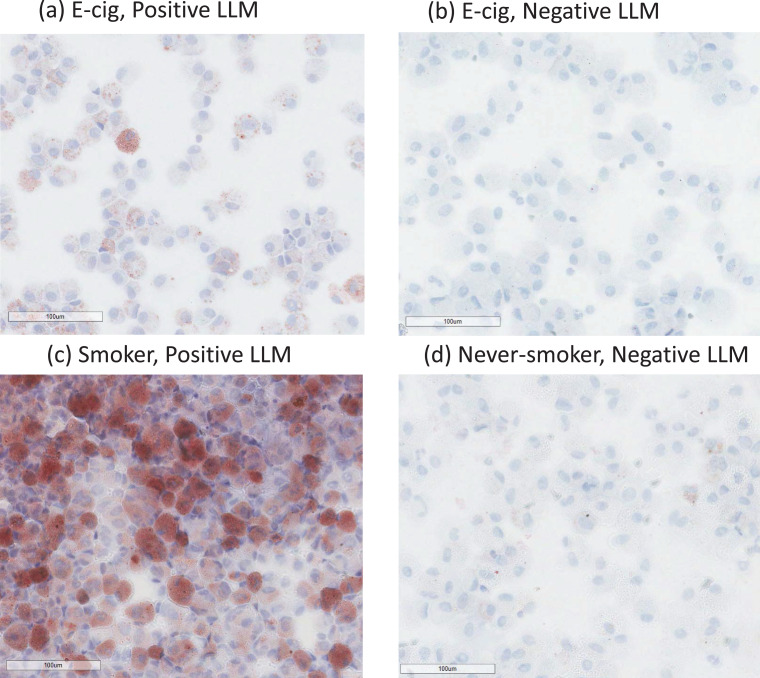
Examples of bronchoalveolar lavage fluid Oil-Red-O stains for LLM showing the presence of macrophages with prominent cytoplasmic lipid accumulation in positive samples. (a) E-cigarette user and (c) smoker; and negative samples with macrophages lacking cytoplasmic lipid: (b) E-cig user and (d) never-smoker. Original magnification x400.

**Fig. 2:**
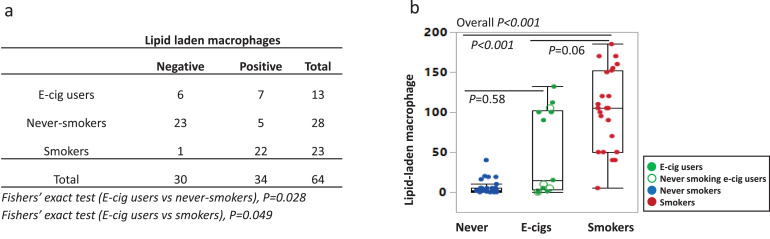
Contingency table and dot plot of lipid-laden macrophages (LLM). (a) LLM were classified as positive (>10 LLM index) and negative. E-cig users were significantly more likely to test positive compared to never-smokers and significantly less likely compared to smokers (Fisher's exact test). (b) Each dot represents one participant, never-smokers in blue (*n* = 28), e-cig users in green (*n* = 13), and smokers in red (*n* = 23). Closed green circles are former smokers; open circles are never smoking e-cig users (*n* = 4). Box plots show medians with interquartile range for never-smokers (blue), e-cig users (green), and smokers (red). Open green circle represents ever-smoking e-cig users. Kruskal–Wallis test was used for overall p-values. Mann–Whitney test was used for two-group comparisons. All statistical tests were performed using batch-removed LLM.

Participant characteristics and LLM results are shown in Table 1**,** and individual participants’ data are included in the **Supplementary Table 1**. Samples were categorized as LLM negative or positive (LLMI>10); example images are shown in Fig. 1. Among 23 smokers, 22 were positive (96%), and most had LLMI levels >50 (87%). Among 27 never-smokers, 5 were positive for LLMI (18%), but at low levels (<50). For e-cig users, 7 of 12 were positive (58%), and 86% of positives were >50. Group differences of LLMI for smokers, e-cig users, and never-smokers were statistically significant (overall *P*<0.001) (Fig. 2 and **Supplementary Table 1)**.

**Table 1:** Characteristics of Study Participants: Bronchoscopy study of never-smokers, e-cig users, and smokers.

	Never-smokers (*n* = 28)	E-cig users (*n* = 13)	Smokers (*n* = 23)	Among groups^1^, p-value	E-cig users vs. Never-smokers^2^, p-value	E-cig users vs. Smokers^2^, p-value	Smokers vs. Never-smokers^2^, p-value
Age(years), mean(SD)	25.5 (3.4)	26.2 (2.9)	27 (3.8)	0.34	0.32	0.95	0.17
Gender				0.02	0.05	1	0.02
Females, N(%)	16 (57%)	3 (23%)	5 (22%)				
Race				0.95	1.00	1.00	0.78
White, N(%)	20 (71%)	10 (77%)	20 (87%)				
Black or African American, N(%)	3(11%)	1 (8%)	1 (4%)				
Asian, N(%)	3(11%)	1 (8%)	1 (4%)				
More than one race, N(%)	1 (4%)	1 (8%)	1 (4%)				
Unknown or not reported, N(%)	1 (4%)	0 (0%)	0 (0%)				
Lipid-laden macrophages, positive, N(%)	5 (18%)	7 (54%)	22 (96%)	<0.0001	0.028	0.0049	<0.0001
Urine, cotinine+3-hydroxycotinine* (nmol/mg creatinine), median(IQR)	0.0 (0.0–0.01)	14.0 (0.8–31.5)	19.5 (5.8–54.4)	<0.0001	0.0003	0.40	<0.0001
Urine, nicotelline* (ng/ml), median(IQR)	–	2 (2–33.9)	1027.7 (231.3–1336.5)	–	–	0.002	–
Urine, anatabine* (ng/ml), median(IQR)	0.1 (0.1–0.1)	0.1 (0.1–1.4)	11.5 (3.9–20.3)	<0.0001	0.0084	0.002	<0.0001
Urine, propylene glycol* (mg/L), median(IQR)	2.1 (0.9–6.0)	25.5 (4.5–55.8)	6.6 (2.5–17.6)	0.006	0.005	0.18	0.04

1 Among groups: never-smokers, e-cig users, and smokers.

⁎ Below quantification limit was replaced by half of the limit of quantification ^1^Kruskal–Wallis test for continuous variables (age and biomarkers), Fisher's exact test for categorical variables (gender and race).

2 Mann–Whitney test for continuous variables (age and biomarkers), Fisher's exact test for categorical variables (gender and race) - no data.

**Fig. 3:**
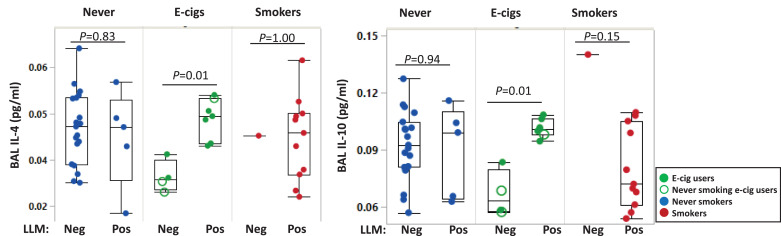
Differences of IL-4 and IL-10 levels by negative and positive lipid-laden macrophages (>10 LLM index) in never-smokers, e-cig users, and smokers in bronchoalveolar lavage fluids. Each dot represents individual never-smokers (blue), e-cig users (green), and smokers (red). The open circles indicate never-smoking e-cig users (smoked<100 cigarettes in their lifetime). Box plots show medians with interquartile range. Mann-Whitney was used for pairwise analyses between negative and positive LLM.

Smoking history, including cigarettes per day and TNE, e-cig use history, gender, smoking- and PG were not correlated with LLMI (all *P*>0.05, Spearman). Nine of 13 e-cig users were former smokers, and most smoked for less than one year. LLMI were not correlated with prior smoking, time since last smoking, cigarettes per day, or inflammatory cell counts in this group (all *P>* 0.05, Spearman). Among e-cig users, LLM (positive and negative) were significantly associated with IL-4 and IL-10 (*P* = 0.01 Fig. 3), but not with the other cytokines(IFN-γ, IL-1β, IL-2, IL-6, IL-8, IL-12p70, IL-13, and TNF-α). Cytokines were not significantly associated with LLM among the never-smokers. For smokers, it was not possible to evaluate these associations because only one smoker had negative LLM. Although reported drug use was an exclusionary criterion, urinary THC was detected in 24% of those tested (13/54), 53% of smokers (9/17), 27% of e-cig users (3/11), and 4% of never-smokers (1/25) (**Supplementary Table 1**). LLMI was not correlated with THC level in the smokers (*P* = 0.22, Spearman), and the numbers of LLM positives were too small to be assessed in the other groups. Subset analyses of subjects confirmed THC-abstinent also showed significant group differences of LLM (*P* = 0.012, data not shown); THC was not a confounder.

## 4 Discussion

EVALI pathology may include the presence of lipid-laden macrophages (foam cells) in the setting of acute fibrinous pneumonitis, diffuse lung injury or organizing pneumonia. There is a current debate as to whether LLM and lipoid pneumonia are a feature of EVALI [1,[3], [4], [5], [6]]. Whether or not LLM are related to EVALI, the recent outbreak emphasizes how little is known about the effects of e-cig use, both short- and long-term. Here, we report for the first time regarding LLM in the lungs comparing healthy smokers, e-cig users, and never-smokers. High levels of LLM were observed in almost all smokers, about half of the e-cig users, and almost none of the never-smokers. In a prior study, consistent with our results, there were more LLM in smokers’ sputum than never-smokers (e-cig users were not included) [13]. Given our observation of high frequency among apparently healthy smokers and e-cig users, LLM are likely not specific to EVALI. In a subset analysis for subjects without detectable THC, the results were similar, indicating that THC was not a confounder (data not shown).

Most of the e-cig users were never-smokers or previously light smokers. The presence of LLM in samples from some of the e-cig users may be related to smoking but more likely represents an e-cig effect unrelated to smoking in a subset of users. In the e-cig users positive for LLM, there were higher levels of the lung inflammatory cytokines IL-4 and IL-10, which were unrelated to smoking [14].

In a recent mouse study, LLM and altered lipid homeostasis were induced by exposure to aerosolized PG/VG from e-cigs, but not by cigarette smoke [15]. Based on this study, it may be that e-cigs induce LLM in humans, but that there is interindividual variation where this effect is only for some users. We assessed some reasons for this, but a limitation of this study is its small size. There also may be interspecies differences for LLM induction. The results of our study being in agreement with a prior study of only smokers and never smokers increases the confidence in the results reported herein [13]. Either way, this finding implies that e-cig use may lead to an increase in LLM through pathways other than from smoking-related disease pathways given its almost universal finding in smokers. In this study, IL-4 and IL-10 were associated with LLM, suggesting further examination is needed to investigate whether LLM may be a marker for disease risk. Phagocytosis of lipids, such as a lipid-rich surfactant, is a normal macrocytic process, but an increase reflects altered lipid homeostasis and a pathological consequence of pathways such as those for pulmonary fibrosis, inflammation, and reduced immunity [16,17].

Among the e-cigs users, a significant association between LLM and lung inflammatory cytokines IL-4 and IL-10 was found, but not other cytokines associated with smoking [14]. Unlike the other cytokines that were tested, IL-4 and IL-10 play a role as an immune suppressor and are required to avoid inflammation and maintain tissue homeostasis [18,19]. IL-4 promotes macrophage activation into the M2 phenotype, and M2 macrophages produce IL-10 [20]. IL-4 induces mucous glycoprotein synthesis and results in the accumulation of mucus in nonciliated epithelial cells; it also promotes goblet cell hyperplasia and the secretion of airway mucus [21]. Apart from the regulatory role and direct action of IL-4 on effector cells in allergic inflammation, this cytokine also appears to have actions relevant to smoking-related diseases. IL-10 is an anti-inflammatory and pro-resolving factor that can work to inhibit pro-inflammatory cytokines [22]. Increased IL-4 is associated with allergic airway response; [23] and both IL-4 and IL-10 alter macrophage lipid metabolism [24]. However, in a separate study from our group, IL-4 and IL-10 were not affected by e-cig use in never-smokers, although the study was of brief duration [25]. Thus, we hypothesize that an association of LLM with increased IL-4 and IL-10 among e-cig users may be a result of responses to compensate for altered lung homeostasis or the hypersecretion of bronchial submucosal glands characteristic of e-cig exposure.

As a cross-sectional study, an important limitation is that a temporal and causal relationship of cigarette and e-cig use with LLM induction cannot be assessed; the association might be the result of unknown confounders. Further, this study was relatively small so that effect modification could not be assessed (e.g., . the association of urinary cotinine and LLMI).

An important strength is that we made measurments by bronchoscopy, directly assessing lung toxicity in healthy participants, comparing smokers, e-cig users, and never-smokers. A further strength is that our study focused on participants in the age group that is particularly affected by EVALI.

Vaping THC oils, but not smoking, are clearly associated with EVALI. Our findings provide evidence that LLM are not a marker of EVALI because they are found in the lungs of healthy smokers. The induction of LLM in smokers and e-cig users might occur through different pathways, given the experimental animal studies cited above. Why only about half of the e-cig users were found to have increased LLMI is unclear, but LLMI may be a marker of e-cig lung toxicity unrelated to smoking. Health effects of e-cigs need to be better understood. In particular, the etiology of LLM in some, but not all e-cig users need further exploration. Also, future research examining EVALI biomarkers should assess the route of THC use.

## Funding sources

Research reported in this publication was supported by funding from the National Cancer Institute of the 10.13039/100000009National Institutes of Health (NIH) (P30 CA016058), the Food and Drug Administration Center for Tobacco Products (CTP) (P50CA180908), the 10.13039/100000050National Heart, Lung, and Blood Institute (R21HL147401), the National Institute on Drug Abuse of the 10.13039/100000009National Institutes of Health (U54DA036105), the 10.13039/100006108National Center For Advancing Translational Sciences (UL1TR001070) and from Pelotonia Intramural Research Funds and the 10.13039/100001299Prevent Cancer Foundation. The content is solely the responsibility of the authors and does not necessarily represent the official views of the NIH or the FDA. None of the funders played a role other than funding.

## Author contributions

PGS and MAS contributed equally to this paper. PGS and MAS made substantial contributions to the conception and design of the work. PGS and MAS interpreted data and were significant contributors in writing the manuscript. MAS analyzed data, and JPM oversaw the statistical data analysis. JLF, TMB, SAR, DYW, RR, TE, and KS all has significant to contribution to the design and evaluation of the data. MDW performed bronchoscopies, edited the manuscript, and provided critical insight into the interpretation of data. PGS and MDW reviewed subjects’ medical and tobacco use history to determine eligibility. DYW and SAR contributed to the sample processing, data collection, and/or assembly. RR and KS evaluated lipid-laden macrophages. As the corresponding author, PGS also had full access to all of the data in the study and had final responsibility for the decision to submit for publication. All authors read, revised, and approved the manuscript.

## Data sharing statement

The data that support the findings of this study are available from the corresponding author, PGS, upon reasonable request.

## Declaration of Competing Interests

PGS has served as an expert witness and consultant in tobacco company litigation on behalf of plaintiffs. TE is a paid consultant in litigation against the tobacco industry and also the electronic cigarette industry and is named on a patent for a device that measures the puffing behavior of electronic cigarette users. The other authors declare that they have no potential conflicts of interest.

## Appendix Supplementary materials

Image, application 1

Image, application 2

**Supplementary Figure 1. Oil red O stain validation.** (a) adipose tissue with red staining of fat globules within adipocytes. (b) Bronchioloalveolar fluid from a current smoker showing brown-red staining of varying in size fat globules some containing dark-black dust deposits. (c) Bronchioloalveolar fluid from a never smoker with acute bronchopneumonia showing a lack of staining in macrophages. (d) Lipid-laden Macrophage Index. Macrophages from bronchioloalveolar lavage showing the variable amount of lipid droplets with a) macrophage negative for lipid, b) low-positive macrophage with less than 50\elsamp #x0025; of the cytoplasm occupied by lipid, and c) high-positive macrophage with more than 50\elsamp #x0025; occupied by lipid; Oil red O stain. Image, application 3
