# Supplementary material for: Lipid laden macrophages and electronic cigarettes in healthy adults
Source: eBioMedicine. 2020 Sep 10;60:102982. doi: 10.1016/j.ebiom.2020.102982 (PMC7494450; doi:10.1016/j.ebiom.2020.102982)
Supplement: Supplementary file 1 [file mmc1.docx]

**Supplement. Medical illness exclusions that may impact biomarker**

1. Any acute illness, after addressing with PIs
2. Autoimmune disorders, after addressing with the PIs
3. Any type of cancer
4. HIV positive or AIDS
5. Cardiovascular diseases such as rheumatic fever, after addressing with the PIs
6. Thyroid diseases, after addressing with PIs
7. Recent severe anemia treated with blood transfusion, or other blood diseases, after addressing with PIs
8. Pulmonary:
9. Acute respiratory diseases
10. Acute bronchitis within 1 year
11. COPD
12. Restrictive lung disease
13. Cancer
14. Chronic bronchitis
15. Asthma within the prior 5 years
16. Pneumonia
17. Tuberculosis
18. Other lung diseases, after addressing with PIs
19. Kidney:
20. Any clinically diagnosed kidney disease, after addressing with PIs
21. Acute urinary tract infections (UTI) treated with antibiotics or steroids within the past 30 days
22. Renal failure
23. Other chronic kidney disease, after addressing with PIs
24. Gastrointestinal:
25. Acute infection
26. Acute Inflammatory bowel disease under treatment with steroids, after addressing with PIs
27. Liver:
28. Any clinically diagnosed liver disease, after addressing with PIs
29. Hepatitis
30. Jaundice
31. Liver cirrhosis or fibrosis
32. Hepatic failure
33. Musculoskeletal:
34. Acute arthritis
35. Chronic arthritis, after addressing with PIs
36. Severe scoliosis (could lead to lung restriction)

**Actual subjects excluded based on reported medical illness**

| **Reasons** | **# of subjects excluded during screening or after enrollment** |
| --- | --- |
| Any acute illness (such as sinusitis, conjunctivitis, recent steroids use) | 9 |
| Cancer | 1 |
| HIV positive or AIDS | 1 |
| Cardiovascular disorders such as rheumatic fever | 1 |
| Thyroid disorders | 1 |
| Acute respiratory disorders | 3 |
| Acute bronchitis within 1 year | 2 |
| COPD | 1 |
| Chronic bronchitis | 1 |
| Asthma within the prior 5 years | 12 |
| Pneumonia | 2 |
| Acute UTI treated with antibiotics or steroids within the past 30 days | 1 |
| Renal failure | 1 |
| Other chronic kidney disease | 1 renal stone |
| Hepatitis | 1 |
| Jaundice | 1 |
| Acute arthritis | 2 |
| Chronic arthritis | 1 used humira and merkaptopurine |
| Recent changes in medications | 2 |
| Multiple medical conditions (separate from above) | 5   - 1(HIV, thyroid disease, UTI used AB) - 4 (details not captured at telephone screening) |
