# Supplementary material for: Lipid laden macrophages and electronic cigarettes in healthy adults
Source: eBioMedicine. 2020 Sep 10;60:102982. doi: 10.1016/j.ebiom.2020.102982 (PMC7494450; doi:10.1016/j.ebiom.2020.102982)
Supplement: Supplementary file 3 — Supplementary Figure 1. Oil red O stain validation. (a) adipose tissue with red staining of fat globules within adipocytes. (b) Bronchioloalveolar fluid from a current smoker showing brown-red staining of varying in size fat globules some containing dark-black dust deposits. (c) Bronchioloalveolar fluid from a never smoker with acute bronchopneumonia showing a lack of staining in macrophages. (d) Lipid-laden Macrophage Index. Macrophages from bronchioloalveolar lavage showing the variable amount of lipid droplets with a) macrophage negative for lipid, b) low-positive macrophage with less than 50\elsamp #x0025; of the cytoplasm occupied by lipid, and c) high-positive macrophage with more than 50\elsamp #x0025; occupied by lipid; Oil red O stain. [file mmc3.pptx]

## Slide 1
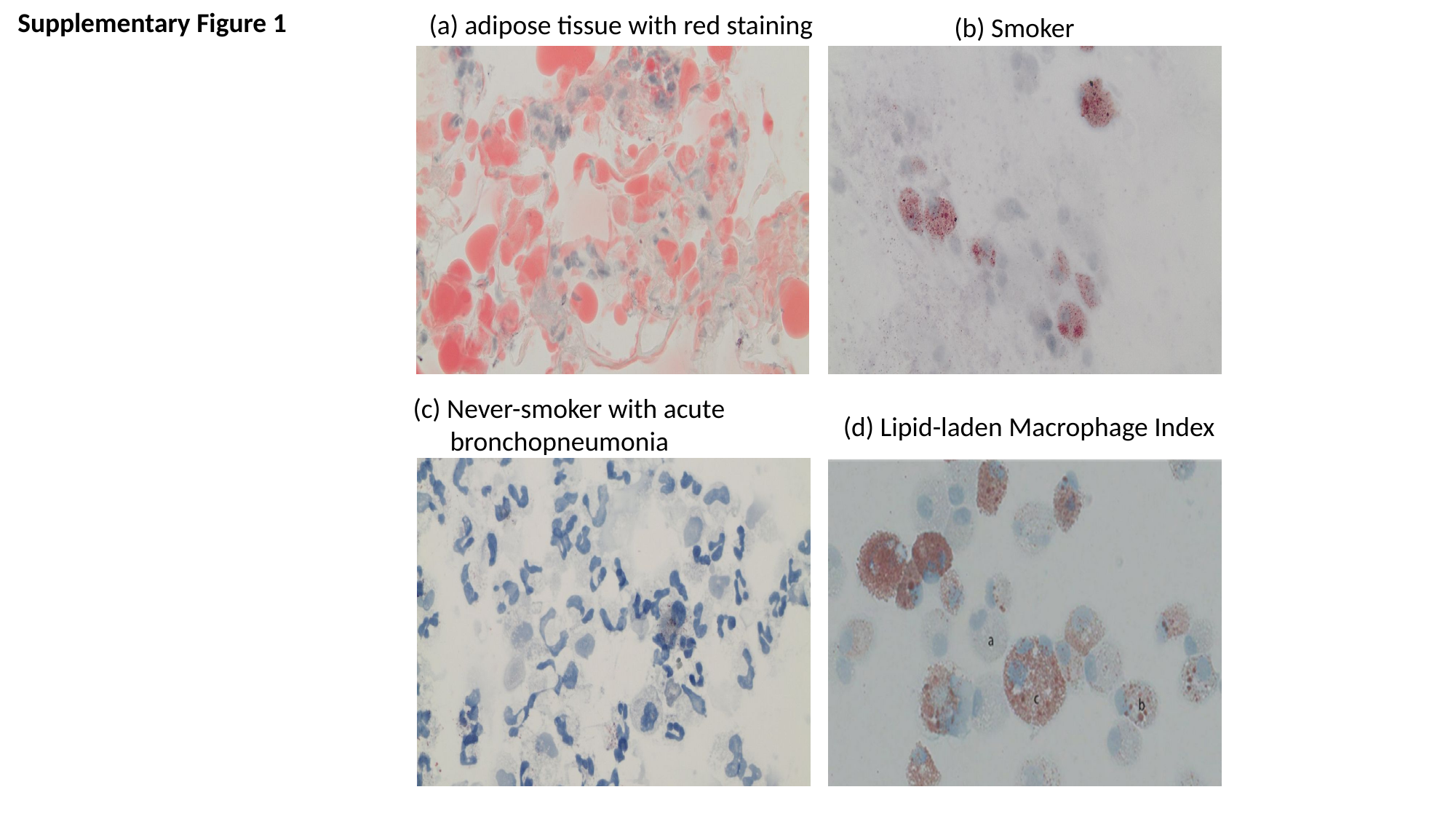

Supplementary Figure 1
(a) adipose tissue with red staining
(b) Smoker
(c) Never-smoker with acute
 bronchopneumonia
(d) Lipid-laden Macrophage Index
